# Elucidating the Gene Expression Signature of Rosai–Dorfman Disease: A Study from the Atlas of Blood Cancer Genomes Initiative

**DOI:** 10.1158/2767-9764.CRC-25-0834

**Published:** 2026-10-30

**Authors:** Sheren Younes, Fadzai Chinyengetere, Veronica Russell, Devang Thakkar, Emily F. Mason, Ridas Juskevicius, Alexandra E. Kovach, Shaoying Li, C. Cameron Yin, Dennis P. O’Malley, Jean L. Koff, David L. Jaye, Cassandra Love, Jennifer R. Shingleton, Dennis Owusu, Ayush Batra, Leonardo P.A. Biral, Elizabeth Thacker, Clay Parker, Lanie Happ, Jennifer Chapman Fredricks, Izidore S. Lossos, Sarah L. Ondrejka, Eric Hsi, Eric Tse, Rex K.H. Au-Yeung, Govind Bhagat, Rebecca J. Leeman-Neill, Amy Chadburn, Magdalena Czader, Yuri Fedoriw, Kikkeri N. Naresh, Sandeep S. Dave, Yasodha Natkunam

**Affiliations:** 1 Stanford University, Palo Alto, California.; 2 Duke University, Durham, North Carolina.; 3 Data Driven Bioscience, Durham, North Carolina.; 4 Division of Hematopathology, Vanderbilt University, Nashville, Tennessee.; 5 Vanderbilt University Medical Center, Nashville, Tennessee.; 6 PathGroup, Nashville, Tennessee.; 7 Department of Hematopathology, The University of Texas MD Anderson Cancer Center, Houston, Texas.; 8 NeoGenomics, Fort Myers, Florida.; 9 Department of Hematology and Medical Oncology, Winship Cancer Institute, Emory University School of Medicine, Atlanta, Georgia.; 10 Department of Pathology and Laboratory Medicine, Emory University, Atlanta, Georgia.; 11 Department of Pathology, University of Miami, Miami, Florida.; 12 Sylvester Comprehensive Cancer Center, Miami, Florida.; 13 Division of Hematology, Department of Medicine, University of Miami, Miami, Florida.; 14 Pathology and Laboratory Medicine Institute, Cleveland Clinic, Cleveland, Ohio.; 15 Mayo Clinic, Rochester, Minnesota.; 16 University of Hong Kong, Hong Kong, Hong Kong SAR, China.; 17 Department of Pathology, University of Hong Kong, Hong Kong, Hong Kong SAR, China.; 18 Department of Pathology, Saint Louis University School of Medicine, SSM Health, Saint Louis, Missouri.; 19 Department of Pathology and Laboratory Medicine, Columbia University Medical Center, New York, New York.; 20 Department of Pathology, Weill Cornell Medical College, New York, New York.; 21 Department of Pathology and Laboratory Medicine, Indiana University, Indianapolis, Indiana.; 22 University of North Carolina, Chapel Hill, North Carolina.; 23 Pathology Program, Translational Science and Therapeutics Division, Department of Pathology, Fred Hutchinson Cancer Center, Seattle, Washington.; 24 Stanford University Medical Center, Palo Alto, California.

## Abstract

Rosai–Dorfman disease (RDD) is a rare histiocytic disorder characterized by the accumulation of large histiocytes in nodal and/or extranodal sites. The underlying biology of RDD and factors that distinguish it from other histiocyte disorders are poorly understood. To better understand the disease biology, we employed whole-transcriptome RNA sequencing (RNA-seq) to study the gene expression profiles of RDD as part of the Atlas of Blood Cancer Genomes (ABCG) initiative. Gene expression profiles of RDD (*N* = 23) were then compared with two other histiocytoses [Langerhans cell histiocytosis (LCH; *N* = 15) and Erdheim–Chester disease (ECD; *N* = 4)], which are most often considered in the clinicopathologic differential diagnosis of RDD. We found that gene expression levels by RNA-seq correlated well with known immunohistochemistry results for diagnostic markers for all three diseases, highlighting the ability to use RNA-based approaches for the diagnosis of these tumors. RDD samples showed enrichment of gene sets related to B cells and the IL27 pathway. In contrast, LCH showed enrichment of gene sets related to resting dendritic cells and the HNF3A pathway. We found significant differences in the composition of the microenvironment, with an enrichment of memory B cells, plasma cells, and CD8 T cells in the RDD microenvironment and enrichment of dendritic cells in the LCH microenvironment. In summary, our study demonstrates unique gene expression signatures in RDD that show overlap as well as distinctive patterns from LCH or ECD. These novel gene expression signatures encompass pathways that could potentially contribute to the pathogenesis and targeted treatment of this rare histiocytic entity.

**Significance:**

To our knowledge, this study is the first to report that protein expression of markers used in the immunohistochemical diagnosis of RDD and other histiocytoses correlates well with gene expression. This finding indicates that RNA-based methods can be used in the clinical diagnosis of RDD. We also demonstrate that RDD shows a unique tumor microenvironment that is rich in immune cells, including B cells, plasma cells, and CD8^+^ T cells.

## Introduction

Rosai–Dorfman disease (RDD) is a rare, clinically heterogeneous histiocytic disorder of unclear pathogenesis. Morphologically, it is characterized by the accumulation of enlarged histiocytes that typically exhibit emperipolesis, in which lymphocytes and other cells become entrapped within histiocytes (1). The classic clinical presentation of RDD is that of massive, bilateral, painless cervical lymphadenopathy, variably accompanied by fever, weight loss, and night sweats. RDD can involve nodal and/or extranodal sites: Nodal RDD predominantly affects lymph nodes in children and young adults, whereas the extranodal form of the disease most often presents in the skin, central nervous system, and bone and soft tissue and affects older adults. Although most patients with RDD are treated with observation or localized therapies, a subset of patients with refractory or disseminated disease suffers from increased morbidity and mortality (1–7).

The diagnosis of RDD can be challenging. Morphologically, nodal RDD shows effacement of the lymph node architecture by a sinusoidal and/or diffuse infiltrate of large histiocytes. Emperipolesis is characterized by histiocytes with intact hematolymphoid cells within vacuoles freely floating in the cytoplasm and is a useful although nonspecific diagnostic feature. Marked sclerosis and an abundance of polytypic plasma cells are usually present in the RDD microenvironment (1–7). The diagnosis of RDD can usually be established on routine hematoxylin and eosin–stained sections with the aid of immunohistochemistry (IHC) to rule out other histiocytoses as well as reactive and inflammatory conditions (4–11). Extranodal RDD closely mimics nodal disease but often shows more prominent reactive lymphoid follicles with germinal centers, increased sclerosis, and fewer histiocytes. In some cases, emperipolesis may be imperceptible, which could pose diagnostic challenges, especially in limited biopsy samples (7).

RDD histiocytes are characteristically positive for S100 protein (S100), CD68, and CD163 but lack CD1a, langerin (CD207), and anaplastic lymphoma kinase (ALK); they also frequently express cyclin D1, OCT2, and PU.1 (8–11). These immunophenotypic markers facilitate the separation of RDD from other histiocytoses, including Langerhans cell histiocytosis (LCH) and Erdheim–Chester disease (ECD), but they require a fair amount of tissue for processing, which can be challenging to obtain. An RNA-based approach for these markers could be cost- and time-effective and require much less tissue, but the degree to which the protein expression matches gene expression in these diseases is completely unknown.

The pathogenesis of RDD remains poorly understood, with conflicting reports on its underlying genetics. Unlike LCH and ECD, in which BRAF V600E mutations are well described (12–14), only isolated cases of BRAF V600E alterations have been reported in RDD (15, 16). Whole-exome and whole-transcriptome sequencing in patients with BRAF V600E–negative non-LCH histiocytoses have identified recurrent kinase fusions involving *BRAF*, *ALK*, and *NTRK1*, along with recurrent mitogen-activated protein kinase (MAPK) pathway alterations, including activating *MAP2K1* and *ARAF* mutations. Importantly, treatment with MEK and RAF inhibitors in patients with *MAP2K1* and *ARAF* mutations has demonstrated clinical efficacy, underscoring the neoplastic nature of non-LCH disorders, including RDD (17). Additionally, mutually exclusive *KRAS* and *MAP2K1* mutations were detected in 30% to 50% of RDD cases in one study, implicating MAPK/ERK pathway activation in its pathogenesis (18); however, no mutations were identified in *ARAF*, *BRAF*, *PIK3CA*, or other genes tested in that study. In contrast, other reported cases of RDD have harbored MAP2K1 L115V (19), KRAS K117N (20), and activating *KRAS* mutations, with a clinical response to cobimetinib therapy (21). Although previously considered a nonneoplastic histiocytic proliferation, the detection of gain-of-function mutations in the MAPK pathway in at least a subset of RDD cases has led to its designation as a clonal neoplasm and its inclusion in the most recent World Health Organization classification of tumors of the hematopoietic and lymphoid tissues (7). There is, however, no defining genetic alteration that has been established to reliably diagnose RDD, particularly in small-volume biopsies or in extranodal sites where fibrosis is prominent and emperipolesis is difficult to identify.

Despite recent advances in understanding the genetic (DNA) underpinnings of histiocytoses, little is known about global gene expression in RDD or how its cellular composition, including the tumor microenvironment, differs from that of related entities. To gain further insights into RDD disease biology, we performed whole-transcriptome RNA sequencing (RNA-seq) in a cohort of clinically well-annotated cases of RDD. We compared the gene expression profiles of RDD with those of LCH and ECD to understand the inherent differences among these entities and to potentially uncover functional cellular pathways that may inform diagnosis, outcome prediction, or new therapeutic approaches. We further examined whether gene expression could potentially play a role in the diagnosis of RDD and other histiocytoses by comparing the expression of well-established protein markers with gene expression.

## Materials and Methods

### Sample collection, library preparation, and sequencing

Study samples were recruited from the Atlas of Blood Cancer Genomes (ABCG) project, an international collaborative effort of 25 clinical sites that aims to develop comprehensive genomic profiles of all blood cancers. The study cohort included 42 diagnostic formalin-fixed, paraffin-embedded (FFPE) tumor biopsies: 23 patients diagnosed with RDD, 15 patients with LCH, and 4 patients with ECD. All diagnostic tissue biopsies were reviewed, and diagnoses were confirmed by expert, panel-based pathology review as part of the ABCG consortium. Additionally, clinical demographic and laboratory data were obtained, where available (Supplementary Table S1). All nucleic acid extractions and sequencing library preparations were performed with Duoseq (Research Kit EPXv3, Data Driven Bioscience) following the manufacturer’s protocol. All libraries underwent RNA-seq (whole transcriptome) on the Illumina platform and were analyzed according to the manufacturer’s recommendations in RNA-only mode (additional information in Supplementary Table S2).

### IHC

IHC data were available through participating institutions and were performed as part of the routine clinical diagnostic workup on FFPE tissue biopsies as per routine protocols. The following markers were tested in most cases: S100, CD68, CD163, cyclin D1, OCT2, PU.1, and CD20.

### RNA-seq analysis

FASTQ files containing RNA-seq reads were trimmed using Trimmomatic (v0.39; ref. 22) in paired-end mode to remove Illumina-specific adapter sequences and low-quality reads. STAR aligner (v2.7.1, RRID: SCR_004463; ref. 23) was utilized for mapping RNA reads to the human genome and transcriptome (GRCh38.p12, with a pseudoautosomal regions mask on chromosome Y). Reads that had at least one primary alignment to the human genome were extracted using SAMtools (v1.13; ref. 24) and additionally filtered based on the manufacturer’s recommendations using GATK (v4.1.2.0, RRID: SCR_001876; ref. 25). Transcript quantification was performed using Salmon (v1.2.1, RRID: SCR_017036; ref. 26), and the transcript-level data were summarized to the gene level using the tximport (v1.14.2, RRID: SCR_016752; ref. 27) library in R (28). The result was a matrix representing raw read counts per gene per patient sample. This matrix was restricted to include only the expression of the protein-coding genes captured by exome sequencing. Genes with missing or uniformly low expression were excluded from further analysis, and the remaining expressed genes were normalized across the entire sample set in R using DESeq2 (v1.40.2, RRID: SCR_015687; ref. 29). DESeq2 was also used to perform all differential expression analyses with default parameters. Tumor microenvironment cell fraction estimation was performed with FARDEEP (RRID: SCR_026704; ref. 30) using the LM22 gene expression signature matrix (31), comprising 547 genes that characterize 22 distinct human immune cell types. Raw gene expression counts with batch correction were used as input for FARDEEP. Gene expression heatmaps were generated using seaborn’s (RRID: SCR_018132; ref. 32) clustermap function applied to a gene-by-sample matrix of robust-scaled log_2_-normalized gene expression values. Gene set enrichment analysis (RRID: SCR_003199; ref. 33) was performed using the Molecular Signature Database (33, 34) with the Benjamini–Hochberg multiple testing correction.

### Disease classifier

To distinguish RDD from LCH and ECD, we trained a supervised classifier using log_2_-normalized RNA-seq expression profiles from 42 tumors, comprising RDD (*n* = 23), LCH (*n* = 15), and ECD (*n* = 4) samples. LCH and ECD disease samples were combined into a non-RDD class. Among the 18,860 quantified genes, the 5,000 genes with the highest variance across samples were retained as candidate features. Classification was performed using elastic net–regularized logistic regression with equal L1 and L2 mixing (L1 ratio = 0.5), class-balanced sample weighting, and the stochastiSAGAc average gradient ameliorated optimization algorithm (35). Performance was evaluated using leave-one-out cross-validation (LOOCV). Within each LOOCV iteration, gene expression features were standardized using parameters estimated exclusively from the training samples, and the regularization strength was selected from 20 candidate values through fivefold cross-validation within the training set. Accuracy was used as the optimization criterion.

Predictions from all held-out samples were aggregated to calculate accuracy; class-specific precision, recall, and F1 score; the confusion matrix; and the area under the receiver operating characteristic curve (AUROC). Candidate marker genes were ranked according to their mean absolute elastic net coefficient across LOOCV models. A minimal five-gene panel comprising *ADGRE1*, *CHI3L1*, *POU2F2*, *GPAT3*, and *CLEC5A* was subsequently evaluated using class-balanced L2-regularized logistic regression (regularization strength = 1) with feature standardization and LOOCV. Statistical evidence of overall class separability was additionally assessed using 1,000 label permutations with a class-balanced L2-regularized logistic regression classifier.

### Statistical analysis

All statistical analyses were performed in R (RRID: SCR_001905). The Mann–Whitney U test was used in any comparisons of continuous variables between independent classes. For differential gene expression analysis, a cutoff of |log_2_(fold change)| > 2 and an adjusted *P* (*P*_adj_) value < 0.05 was applied.

## Results

### Clinical and laboratory features of patients with RDD

The RDD cohort consisted of 23 patients (Table 1). Median age at diagnosis was 54 years (range, 1–78 years) in the 19 patients for whom a documented age was available. Eighty-nine percent of patients (17/19) were adults, defined as age 18 or greater, and the majority were female (64%; Table 1). Nodal-only disease (29%) was the least prevalent, with most patients presenting with either extranodal disease alone or coexisting nodal and extranodal disease. Concurrent comorbidities in the cohort of patients with RDD included autoimmune, inflammatory, and other malignant conditions. In 21 RDD cases with documented IHC analysis, all samples were positive for S100. Most cases tested were also positive for CD68, PU.1, and OCT2, with the latter known to be specific for RDD. Representative histology and IHC staining of RDD are shown in Supplementary Fig. S1.

**Table 1.** Baseline clinical and demographic data of 23 patients with RDD.

Characteristic	*N* (number of samples with data available)	Value^a^
Age at diagnosis	19	54 (32, 68)
Patient gender	22	​
Female	​	14 (64%)
Male	​	8 (36%)
Self-reported race	10	​
White	​	5 (50%)
Black	​	4 (40%)
Asian/Pacific Islander	​	1 (10%)
Presentation site	21	​
Extranodal	​	12 (57%)
Nodal	​	6 (29%)
Both	​	3 (14%)
S100	21	​
Positive	​	21 (100%)
CD68	11	​
Strong positive	​	8 (73%)
Medium positive	​	2 (18%)
Weak positive	​	1 (9.1%)
Cyclin D1	9	​
Strong positive	​	5 (56%)
Weak positive	​	4 (44%)
PU.1	10	​
Strong positive	​	9 (90%)
Weak positive	​	1 (10%)
OCT2	10	​
Strong positive	​	9 (90%)
Weak positive	​	1 (10%)

NOTE: The middle column denotes the number of samples with data available for the indicated characteristic.

a Median (Q1, Q3); *n* (%).

### Differential gene expression profiles distinguish RDD cases

**Figure 1.**
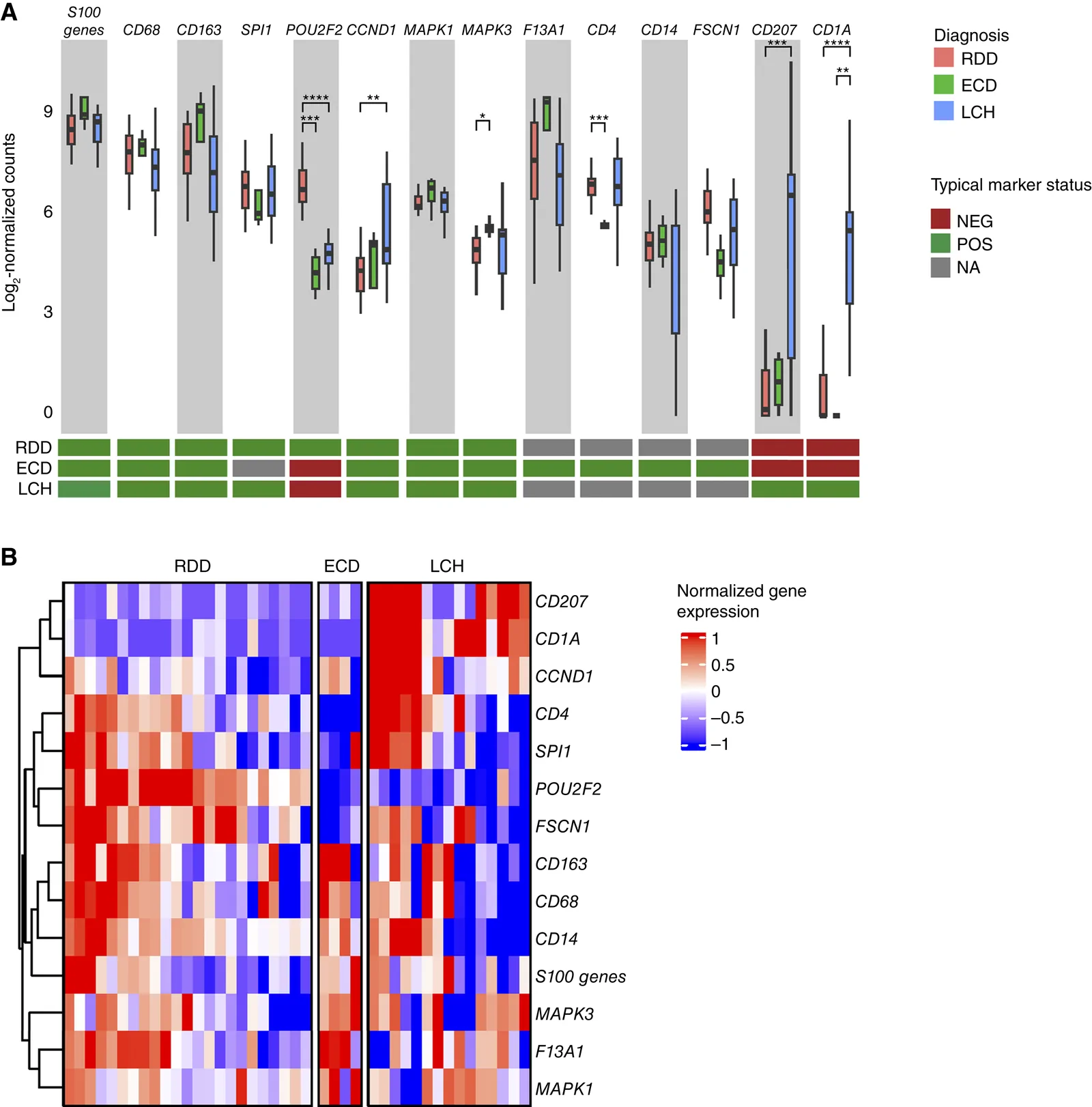
RNA expression of immunohistochemical markers typically used in the diagnosis of histiocytic neoplasms. **A,** Gene expression (represented as log_2_-normalized counts) correlates with typical immunohistochemical marker status (bottom). *, *P* < 0.05; **, *P* < 0.01; ***, *P* < 0.001; ****, *P* < 0.0001 by Mann–Whitney U test. **B,** Heatmap showing normalized gene expression of the protein markers used in the diagnosis of RDD, LCH, and ECD. S100 gene expression is represented as the combined expression of 21 S100 genes shown in Supplementary Fig. S2.

We first compared the expression of histiocytic surface marker genes (S100, CD68, and CD163) with clinical results by IHC. All RDD, LCH, and ECD samples showed high gene expression of these markers (Fig. 1A and B). Of note, S100 gene expression in Fig. 1 represents combined expression from the multiple S100 genes, as shown in Supplementary Fig. S2. Gene expression of *POU2F2*, which encodes the protein OCT2 that is used to distinguish RDD from other histiocytoses, was significantly elevated in RDD compared with both LCH and ECD (*P* < 0.05; Fig. 1A and B). Furthermore, gene expression of *CD1A* and *CD207* was significantly higher in LCH compared with RDD (*P* < 0.05, Fig. 1A and B), which recapitulates the immunophenotyping pattern used to diagnose LCH and separate LCH from RDD. These results indicate strong agreement between protein and gene expression for most markers used in the diagnosis of RDD. The heatmap in Fig. 1B shows the trend in gene expression between the different histiocytic neoplasms but also shows the heterogeneity that exists within the individual samples, even within the same disease type.

**Figure 2.**
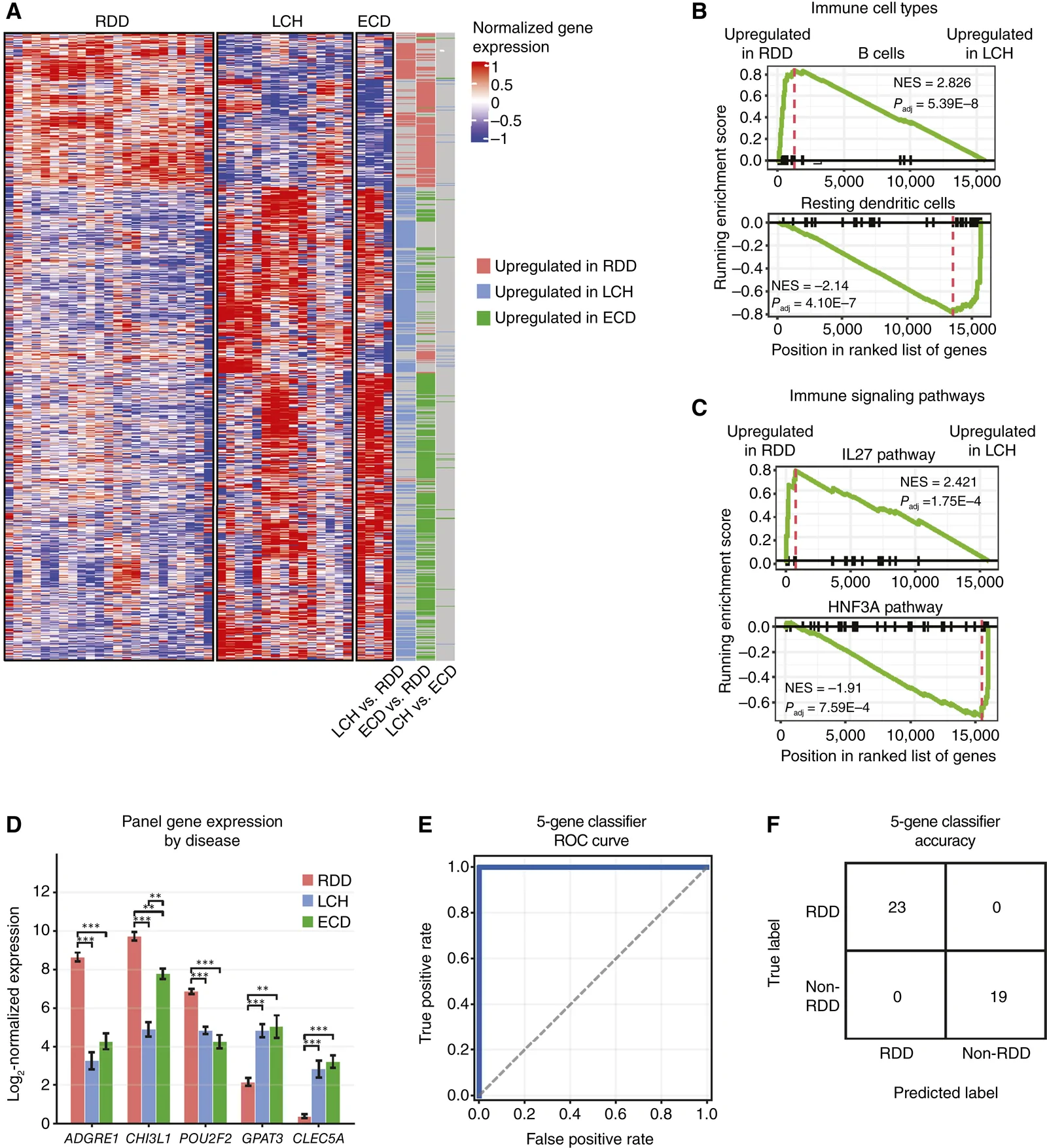
Differential gene expression distinguishes RDD from LCH and ECD. **A,** (Left) Individual normalized gene expression profiles of RDD, LCH, and ECD, respectively. (Right) Differential gene expression profiles of LCH vs. RDD, ECD vs. RDD, and LCH vs. ECD, respectively. Genes upregulated in each diagnosis are color-coded as indicated to the right. **B** and **C,** Gene set enrichment analysis showing upregulation of B cell genes (NES = 2.83, *P*_adj_ = 5.39 × 10^−8^) and IL27 pathway genes (NES = 2.42, *P*_adj_ = 1.75 × 10^−4^) in RDD and resting dendritic cell genes (NES = −2.14, *P*_adj_ = 4.10 × 10^−7^) and HNF3A pathway genes (NES = −1.91, *P*_adj_ = 7.59 × 10^−4^) in LCH. *P* values are adjusted by Benjamini–Hochberg correction. **D,** Each gene in the minimal marker panel exhibits significantly different expression in RDD (*N* = 23) compared with both LCH (*N* = 19) and ECD (*N* = 4). Shown are the mean and SE of log_2_-normalized expression scores. **, *P* < 0.01; ***, *P* < 0.001 by Mann–Whitney U test. **E,** Receiver operating characteristic (ROC) curve of the 5-gene classifier in distinguishing RDD (*n* = 23) from non-RDD (LCH and ECD, *n* = 19) cases. AUROC = 100%. **F,** Confusion matrix showing 100% accuracy of the 5-gene classifier in distinguishing RDD from non-RDD cases.

We used DESeq2 to analyze genes differentially expressed between the three diagnoses. Upon analysis of the differential gene expression profiles of RDD, LCH, and ECD, we observed that LCH and ECD had more similar gene expression profiles, whereas RDD had a unique profile compared with the other two histiocytoses (Fig. 2A; Supplementary Fig. S3). However, the limited number of ECD samples available makes comparisons involving this entity exploratory in nature. We applied principal component analysis and found that diagnosis, institution, and biopsy site were not primary confounders of gene expression (Supplementary Fig. S4).

Compared with LCH, RDD showed strong upregulation of genes related to B cells [normalized enrichment score (NES) = 2.826, *P*_adj_ = 5.39 × 10^−8^] and the IL27 pathway (NES = 2.421, *P*_adj_ = 1.75 × 10^−4^, Fig. 2B and C; Supplementary Table S3). Conversely, LCH showed upregulation of genes related to resting dendritic cells (NES = −2.14, *P*_adj_ = 4.10 × 10^−7^) and the HNF3A pathway (NES = −1.91, *P*_adj_ = 7.59 × 10^−4^, Fig. 2B and C; Supplementary Table S3). No gene set enrichment studies were done for ECD, given the small sample size. To further confirm enrichment for B cells in RDD, we stained 17 RDD and 4 LCH cases (unrelated cases from an independent cohort) for CD20 and found that RDD cases were enriched in CD20^+^ B cells compared with LCH (*P* = 0.0036 by *t* test, Supplementary Fig. S5).

We next sought to build a classifier that could accurately distinguish between histiocytic diagnoses based on gene expression profiles. Due to the limited number of ECD cases, we built our logistic regression classifier to distinguish RDD (*N* = 23) versus non-RDD (*N* = 19) cases. We utilized an elastic net penalized logistic regression with a 1:1 L1-to-L2 penalty. The combined penalty regularizes against overfitting and performs implicit feature selection. We used LOOCV with nested fivefold cross-validation over 20 candidate regularization strength parameter values inside each training fold to tune the regularization strength. We performed standardization and lambda selection on the 41-sample training split only and then applied it to the held-out sample so no information from the test case leaked into preprocessing, hyperparameter choice, or model fitting.

The resulting classifier achieved 100% accuracy and an AUROC of 1. We quantified the uncertainty around this estimate and confirmed that it does not arise by chance. The accuracy of 100% (42/42) corresponds to a Clopper–Pearson 95% confidence interval of (0.92–1; i.e., performance consistent with at most ∼8% misclassification at the population level). To verify that the separation is not an artifact of small sample size, we performed a label-permutation test (1,000 permutations) under the same LOOCV scheme using a conservative fixed-penalty L2 linear classifier. Compared with the permutation null distribution, our model’s performance was significantly higher (empirical permutation *P* = 0.001). Additionally, no permutation matched the observed accuracy for this estimator (92.9%). Therefore, the class separation of our fitted model is statistically unlikely to reflect chance structure.

We then sought to establish the feasibility of translating our classifier to the clinic. We constructed a gene marker panel that might be suitable for clinical assays such as quantitative PCR or NanoString. Across our folds, we identified a set of five genes (*ADGRE1*, *CHI3L1*, *POU2F2*, *GPAT3*, and *CLEC5A*) that had the highest mean absolute value across all folds in the elastic net logistic regression model training protocol. We verified that each of these genes had significantly different expression between RDD and non-RDD cases (*P* < 0.01, Fig. 2D). We found that an elastic net logistic regression model trained with only these genes as features also achieved perfect classification accuracy (AUROC = 1, accuracy = 100%, Fig. 2E and F). This set of genes could therefore be the basis of a clinical gene panel for the differential diagnosis of RDD versus other histiocytic disorders.

### Tumor microenvironment deconvolution reveals differences in immune cell populations

**Figure 3.**
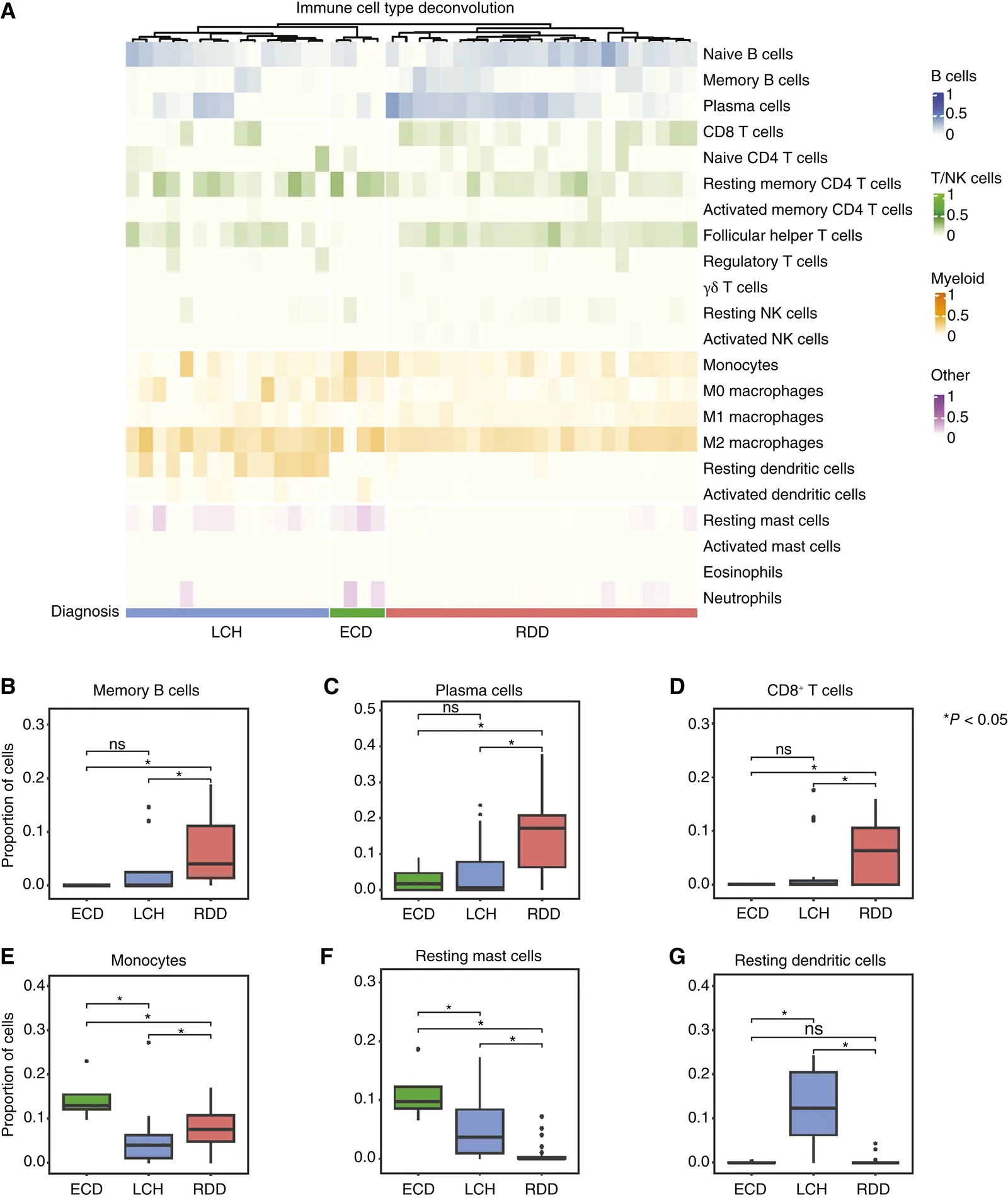
Tumor microenvironment of RDD, LCH, and ECD: (**A**) Cell type deconvolution in RDD, LCH, and ECD. The proportion of each cell type is indicated by the color scales to the right. **B,** Cell types are in blue, T/NK cell types are in green, myeloid cell types are in orange, and other immune cell types are in purple. **B–G,** Box plots showing expression of memory B cells (**B**), plasma cells (**C**), CD8^+^ T cells (**D**), monocytes (**E**), resting mast cells (**F**), and resting dendritic cells (**G**) in the different histiocytic neoplasms, extrapolated from the cell deconvolution map. ns, not significant; *, *P* < 0.05 by Mann–Whitney U test.

We also examined the microenvironment using cell type deconvolution of the expression profiles. We found that the RDD microenvironment was significantly enriched for memory B cells, plasma cells, and CD8 T cells compared with ECD and LCH (*P* < 0.05, Fig. 3A–D; Supplementary Table S4). In contrast, the LCH microenvironment was more enriched in dendritic cells compared with other histiocytic subtypes (*P* < 0.05, Fig. 3G). Although the sample size was small, the ECD microenvironment was enriched for monocytes and resting mast cells (*P* < 0.05, Fig. 3E and F).

## Discussion

Our study is part of the ABCG initiative, which included case collections from a global consortium, with standardized morphologic and immunohistochemical evaluation performed by expert hematopathologists (J.R. Shingleton; in preparation). The cases underwent gene expression profiling to define the molecular differences between RDD and other histiocytic disorders, namely LCH and ECD. Our approach enabled the discovery of distinct gene expression profiles in RDD cases compared with LCH and ECD, as well as the identification of clear differences in tumor microenvironment composition according to disease types.

Until recently, RDD was considered a nonneoplastic histiocytic proliferation; however, the detection of genomic alterations in MAPK pathway genes in a subset of cases has led to its classification as a clonal neoplastic entity (7). Conflicting results, possibly attributed to differences in molecular detection methods, have been noted, particularly with regard to *BRAF* alterations among histiocytoses (15–18). It is unclear whether there are two subtypes of RDD: one reactive and a second clonal neoplastic entity with recurrent activating MAPK pathway alterations (36). Currently, RDD is a clinicopathologic diagnosis with significant heterogeneity in its clinical presentation and disease course, as well as a variable molecular profile that warrants further investigation.

Consistent with prior knowledge about immunohistochemical marker expression in histiocytic disorders, all RDD, LCH, and ECD samples showed high expression of genes encoding the S100, CD68, and PU.1 markers. As expected, gene expression of *POU2F2*, which encodes the OCT2 protein, was significantly elevated in RDD in contrast to LCH and ECD, and the *CD1A* and *CD207* genes were highly expressed in LCH compared with RDD. Although there was considerable heterogeneity in gene expression among the different histiocytic neoplasms, our data nevertheless indicate that gene expression agrees well with protein expression and could be used in place of protein expression in some circumstances.

Our analysis of gene expression profiles further revealed that RDD has a unique gene expression signature compared with LCH and ECD. We used this differential gene expression to build a logistic regression classifier capable of distinguishing RDD from non-RDD cases. We further derived a five-gene panel to demonstrate the feasibility of translating the classifier to clinical use. In general, the five genes in our panel are associated with immune functions and the regulation of the tumor microenvironment. As noted, *POU2F2* encodes the RDD marker OCT2. The genes *ADGRE1*, *CLEC5A*, and *CHI3L1* have all been associated with the activation of tumor-associated macrophages and subsequent cancer cell growth and metastasis in colon cancer (37–40). *GPAT3* has also been noted to contribute to chemoresistance in colon cancer by inhibiting immunogenic cell death of tumors and facilitating CD8 T-cell exhaustion (41). Due to the rarity of these diseases, we did not have sufficient cases for an independent validation cohort. Therefore, validation of the classifier will need to be the subject of future study. If adopted clinically, an assay based on this gene panel would be more efficient in terms of time, cost, and biopsy material than the current clinical standard of IHC staining.

RDD demonstrated strong enrichment of gene sets related to B cells and the IL27 pathway, whereas LCH showed enrichment of gene sets related to resting dendritic cells and the HNF3A pathway. Aberrant IL27 expression has been implicated in multiple autoimmune and inflammatory diseases, yet its role in histiocytic disorders remains unexplored. In our study, we report for the first time increased IL27 abundance in RDD, concomitant with increased B cells.

In contrast to IL27, which is predominantly associated with autoimmune/inflammatory disorders, LCH was enriched in gene sets related to the HNF3A/FOXA1 pathway, which is primarily associated with neoplastic disorders. IL27 and HNF3A underscore the distinct and divergent biology between RDD and LCH, while also pointing to HNF3A as a potential therapeutic focus, although its association with BRAF alterations requires further investigation. Further studies will also be needed to elucidate its impact in LCH on a cellular level.

Prior studies have demonstrated that the pathologic histiocytes of LCH are of myeloid dendritic cell origin, arising from the bone marrow rather than from normal cutaneous dendritic cells known as “Langerhans cells” (42, 43). The most highly upregulated genes in LCH are those of early myeloid cell development, and *BRAF* mutations are present in >50% of biopsies from patients with LCH (12). The histogenesis of ECD and LCH is remarkably similar, and in fact, some patients develop both diseases, reflecting their shared origin from myeloid dendritic cells. In ECD, however, the developmental pathway is skewed toward the macrophage lineage, and although we have a small sample size, our tumor microenvironment studies show significant gene expression of macrophages in ECD compared with the other histiocytoses. Similar to LCH, approximately 80% of patients with ECD harbor mutations in the RAS/RAF/MEK/ERK cellular signaling pathway (44), with frequent BRAF V600E mutations (13).

To the best of our knowledge, this is the first study to perform cell type deconvolution analyses in RDD, LCH, and ECD. Both gene expression and cell type deconvolution studies further support the notion that RDD is biologically distinct from LCH and ECD and align with the known morphologic and immunohistochemical features of these entities.

In conclusion, our study demonstrates the utility of whole-transcriptome RNA-seq in distinguishing histiocytic neoplasms, particularly RDD from LCH and ECD. However, our study is limited by the relatively small sample size, especially for ECD cases. Comparisons involving ECD cases in this study are necessarily exploratory in nature. Future studies with larger cohorts are needed to validate these findings, further explore the heterogeneity of RDD, and better define the cellular microenvironment composition, particularly using spatial proteomics and transcriptomics approaches.

## Supplementary Material

Supplementary Figure S1 Figure S1. Histologic and immunophenotypic features of Rosai-Dorfman disease.

Supplementary Figure S2 Figure S2. RNA expression of the S100 gene isoforms.

Supplementary Figure S3 Figure S3. Differential gene expression of the histiocytic neoplasms expressed in Venn-diagram form.

Supplementary Figure S4 Figure S4. Depiction of principal components analysis (PCA) of gene expression stratified by diagnosis (A), institution (B), and biopsy site (C).

Supplementary Figure S5 RDD cases are enriched in CD20+ B cells compared to LCH. Representative CD20 immunohistochemistry images of RDD (A) and LCH (B). Both images at 150X magnification. (C) The mean percentage of CD20+ B cells was calculated in n=17 slides of RDD cases and n=4 slides of LCH cases and was significantly higher in RDD (p=0.0036, t-test).

CRC-25-0834_supplementary_table_ST1-ST4 CRC-25-0834_supplementary_table_ST1-ST4

## Data Availability

The sequencing data are available in the European Genome Archive at EGAD50000002001, EGAD50000002002, EGAD50000002003, EGAD50000002004, EGAD50000002005, and EGAD50000002006. All other data generated in this study are available within the article and its supplementary files or from the corresponding author upon request.
